# Future Drug Targets in Periodontal Personalised Medicine—A Narrative Review

**DOI:** 10.3390/jpm12030371

**Published:** 2022-02-28

**Authors:** Pradeep Kumar Yadalam, V. Kalaivani, Hammam Ibrahim Fageeh, Wael Ibraheem, Manea Musa. Al-Ahmari, Samar Saeed Khan, Zeeshan Heera Ahmed, Hesham H. Abdulkarim, Hosam Ali Baeshen, Thodur Madapusi Balaji, Shilpa Bhandi, A. Thirumal Raj, Shankargouda Patil

**Affiliations:** 1 Department of Periodontics, Saveetha Dental College and Hospitals, Saveetha Institute of Medical and Technical Sciences, Saveetha University, Chennai 602117, India; pradeepkumar.sdc@saveetha.com; 2 Department of Periodontics, SRM Kattankulathur Dental College & Hospital, SRM Nagar, Chennai 603203, India; kalaivav@srmist.edu.in; 3 Department of Preventive Dental Sciences, College of Dentistry, Jazan University, Jazan 45142, Saudi Arabia; hafageeh@jazanu.edu.sa (H.I.F.); wibraheem@jazanu.edu.sa (W.I.); 4 Department of Periodontics and Community Medical Science, College of Dentistry, King Khalid University, Abha 61421, Saudi Arabia; abudanahmm@gmail.com; 5 Department of Maxillofacial Surgery & Diagnostic Sciences, Division of Oral Pathology, College of Dentistry, Jazan University, Jazan 45142, Saudi Arabia; samarkhan8@gmail.com; 6 Department of Restorative Dental Sciences, College of Dentistry, King Saud University, Riyadh 11451, Saudi Arabia; aheera@ksu.edu.sa; 7 Advanced Periodontal and Dental Implant Care, Missouri School of Dentistry and Oral Health, A. T. Still University, St. Louis, MO 63104, USA; heshamabdulkarim@atsu.edu; 8 Department of Orthodontics, College of Dentistry, King Abdulaziz University, Jeddah 21589, Saudi Arabia; drbaeshen@me.com; 9 Tagore Dental College and Hospital, Chennai 600127, India; tmbala81@gmail.com; 10 Department of Restorative Dental Sciences, Division of Operative Dentistry, College of dentistry, Jazan University, Jazan 45142, Saudi Arabia; shilpa.bhandi@gmail.com; 11 Department of Oral Pathology and Microbiology, Sri Venkateswara Dental College and Hospital, Chennai 600130, India; thirumalraj666@gmail.com

**Keywords:** drug targets, gene therapy, host modulation, personalized medicine, proteomics, periodontal diseases, nanotechnology, vaccines

## Abstract

Periodontal disease is an infection-driven inflammatory disease characterized by the destruction of tooth-supporting tissues. The establishment of chronic inflammation will result in progressive destruction of bone and soft tissue changes. Severe periodontitis can lead to tooth loss. The disease has complex pathogenesis with an interplay between genetic, environmental, and host factors and pathogens. Effective management consists of plaque control and non-surgical interventions, along with adjuvant strategies to control inflammation and disrupt the pathogenic subgingival biofilms. Recent studies have examined novel approaches for managing periodontal diseases such as modulating microbial signaling mechanisms, tissue engineering, and molecular targeting of host inflammatory substances. Mounting evidence suggests the need to integrate omics-based approaches with traditional therapy to address the disease. This article discusses the various evolving and future drug targets, including proteomics, gene therapeutics, vaccines, and nanotechnology in personalized periodontal medicine for the effective management of periodontal diseases.

## 1. Introduction

Periodontal diseases consist of a cluster of pathological conditions initiated by a complex microbial community causing inflammation within the supporting tissues of the teeth leading to attachment and tooth loss. Severe periodontitis is one of the leading causes of tooth loss worldwide. Periodontitis is thought to affect 20–50% of the world’s population and is the 11th most prevalent disease globally [1]. The main etiology of periodontal diseases can be attributed to bacterial biofilm [2]. Several hosts and environmental factors such as genetic predisposition, lifestyle, oral hygiene, systemic conditions, and stress [3] contribute to the onset and progression of periodontitis [4]. Several drugs can modify the inflammatory and immunological response of the periodontal tissues to bacterial plaque [5]. Drugs can provide effective periodontal treatment, low risk, and are affordable. Specialized drug delivery to precise drug targets has the potential to suppress and eradicate periodontal pathogenic microflora for periodontal disease management and treatment [6]. This paper focuses on non-specific etiologies as there is a wealth of evidence in the literature for microbial bacterial drug targets in periodontitis [7]. This paper gives an overview of the current state of research and recent advances in identifying drug targets for the treatment of personalized periodontal disease.

## 2. Host Modulation

Periodontal diseases are a collective term used to describe chronic inflammatory conditions of the gingiva, periodontal ligament, and bone supporting teeth. Although a vast body of research has unequivocally established the role of pathogenic bacteria in dental plaque, the pathogenesis of periodontal diseases remains nebulous [8]. The most common approach to treating periodontal diseases aims at reducing or modifying the bacteria in the dental biofilm through mechanical means [9]. In subjects susceptible to periodontitis, the host response may be disproportional, dysregulated, and destructive [10]. In such cases, modulating the host response has become an alternative therapeutic approach through modification or manipulation of the immune response to decrease or suppress undesired reactions [9,11,12]. It restores the balance between the pro and anti-inflammatory mediators [13]. Studies have suggested that host modulation along with scaling and root planning has helped in the treatment of Grade B Stage 2 periodontitis [14,15].

Host modulation can be accomplished through several approaches. Systemic and local pharmacological drugs can help in reducing the disease progression. The most commonly used drugs are antibiotics, such as tetracyclines, in non-antimicrobial doses to inhibit collagenolytic activities and activities of neutrophils and osteoclasts [16,17]. The anti-inflammatory drugs most commonly used are NSAIDs (non-steroidal anti-inflammatory drugs) which block the cyclooxygenase pathway of arachidonic acid metabolism [18]. Bone sparing drugs such as bisphosphonates bind to hydroxyapatite crystals and interfere with osteoclastic activity preventing bone loss [19]. Inhibition of inflammatory mediators such as nitric oxide, interleukins, and MMPs (matrix metalloproteinases) has also shown an effect against bone resorption [20]. These drugs can improve the periodontal treatment outcome. However, the results may decline after drug withdrawal as these drugs have adverse gastrointestinal and renal side effects that preclude their prolonged use [21,22,23].

Anti-cytokine therapy focuses on blocking the action of proinflammatory cytokines such as interleukins and tumor necrosis factors. Animal studies have found that using receptor antagonists can block the action of inflammatory cytokines [24,25]. However, there are concerns regarding the potential adverse effects of anti-cytokine therapy on immunity.

Specialized pro-resolving lipid mediators have shown the ability to protect against periodontitis in animal studies [26,27,28]. Resolvins act by preventing further neutrophil recruitment. Dietary supplementation of Omega-3 polyunsaturated fatty acids, which are precursors of resolvins and protectins, may be a viable approach for treating periodontal disease [29,30]. When combined with low doses of aspirin, they have been shown to increase the production of resolvins [31]. Experimental studies show that resolvins can regulate inflammation and periodontitis, allowing for the regeneration of damaged bone [32]. Resolvins, rather than suppressing inflammatory processes, drive cellular processes towards health through multiple receptor-mediated events [33].

More recent approaches to host modulation involve targeting the cell pathways important for the gene expression of the inflammatory mediators [9]. In periodontal diseases, the most important pathways are mitogen-activated protein kinase(MAPK), nuclear factor kappa B(NF-ĸB) [34], and Janus tyrosine kinase-signal transducer and activator of transcription (JAK/STAT) [35]. 

Several compounds have potential host modulation actions [9,36]. SD-282 targets the p-38 MAPK pathway and helps in osteoclastogenesis and reduction of alveolar bone resorption in animal models [37,38]. Compound SC-409 reduced streptococcal-induced arthritis, joint swelling, and bone destruction by targeting the p-38 gene [39]. Other compounds which also target the p-38 gene to reduce inflammation include SB-242235 [40], AW-814141 [41], BIRB-796 [42], VX-702 [43], and VX-745 [44]. SP600125 targets the JNK pathway and leads to a reduction in the level of TNF-α, INF-ɤ, IL-6, COX-2, and MMPs and also reduced joint destruction [45]. FR180204 targets the extracellular signal-regulated kinase (ERK) and is effective against mouse collagen-induced arthritis [46]. BMS-345541 targets the NF-ĸB pathway, which was found to decrease both synovial inflammation and joint destruction in the collagen-induced arthritis model in mice [47]. 

The complement system of a component of the innate immune system is tasked with immunosurveillance, triggering inflammation, opsonization, clearing apoptotic cells and immune complexes [48]. However, dysregulation or excessive activation of this system can amplify inflammation [49]. Complement modulation may apply in periodontal therapeutics through inhibition of downstream inflammatory mediators. Animal studies have shown that mice genetically deficient in the complement receptors did not develop inflammation or alveolar bone loss in cases of induced periodontitis [50,51]. AMY-101, a peptide inhibitor, has shown promise in early trials in reducing periodontal disease parameters and reversing periodontitis [52,53]. Locally applied complement modulation could effectively intervene in oral inflammatory diseases such as gingivitis and periodontitis.

The complement system may act synergistically with Toll-like receptor cells to enhance the production of inflammation promoters such as interleukin-17 to worsen bone loss and periodontitis [54]. Multiple toll-like receptors exist in the human body, which can sense molecular patterns associated with pathogens and activate pathways that unleash proinflammatory cytokines and chemokines [55,56,57]. Interventional strategies that target signaling in toll-like receptors, such as the use of transplanted mesenchymal stem cells or toll-like receptor agonists, may have the potential to modulate immune responses in periodontal disease [58].

To summarize, every host-modulation approach is beyond the scope of this review, as most are still in the clinical trial stages. The most recent promising rational targeted approaches have been enumerated. Locally administered host modulation therapies to target specific pathways have a promising future in periodontal therapy.

## 3. Gene Therapy

Human gene therapy involves modifying or manipulating the expression of a gene to alter the biological properties of cells. Genetic modification of cells can be used to control periodontal diseases and help to reconstruct damaged periodontal apparatus in periodontal tissue engineering [59,60]. Gene-based therapeutics would carry a gene into progenitor tissues, signaling them to differentiate into a phenotype favoring regeneration and repair, transforming a defect [61]. Transfering the desired DNA into a specific cell would introduce specific protein synthesis in the cells. Animal studies have found that bubble liposomes can efficiently deliver plasmid DNA into the gingiva, paving the way for targeted tissue therapy [62].

Gene therapy finds application in treating periodontal diseases in the form of periodontal vaccination, approach to biofilm antibiotic resistance, alveolar remodeling, control disease progression, and treatment disease progression. 

Gene-based therapy principles in conjunction with tissue engineering can help regenerate tooth-supporting structures. Gene Activated Matrix (GAM) technology combines gene therapy with tissue engineering to deliver cytokines and growth factors as plasmid genes. GAM systems act as a local gene depot maintaining gene expression and promoting growth factors within the microenvironment, leading to tissue regeneration [63]. This technology has already seen success in skin and cartilage repair [64], and animal studies show promising results for this nonviral gene delivery system [65,66].

Gene therapy-based periodontal tissue engineering treatments have immense potential for tissue regeneration through stimulation of signaling pathways linked to cell proliferation and differentiation. However, they may be limited by the number of cells or molecules that can be introduced into a defect area for correction, or by scaffold degeneration and extracellular matrix deposition that could cause fibrosis. Cell sheet engineering is a technology that can overcome any problems associated with artificial scaffolds. Scaffold-free cells can be harvested with the cellular proteins and extracellular matrix intact. Periodontal ligament cell sheet culture is now undergoing clinical trials [67].

Gene therapy uses various vectors for the delivery of genes that encode growth factors which stimulate the regeneration of cells of the periodontium. Tissue engineering helps in the regeneration of tissues such as TGF-β, BMP-2,6,7,12, bFGF, VEGF, and PDGF. The vectors can be used at the site by in vivo or ex vivo techniques. Gene transfer can be performed ex vivo and in vivo. Ex vivo gene transfer is done in a cell culture environment where the cells carrying the foreign gene are carried back to the host cell. Research indicates that cells types such as non-osteogenic fibroblast, myoblasts, osteoblasts can help express genes such as BMP-7 and BMP-9 on being infected with an adenoviral vector. These cells then undergo differentiation into bone-forming cells [68].

Platelet-derived Growth Factor (PDGF) is a chemotactic and mitogenic activator for osteoblasts, periodontal ligament cells, and gingival fibroblasts [69]. Different forms of PDGF have been known to enhance wound healing through chemotaxis and mitogenesis [70]. However, the growth arrest of the gene encoding for PDGF leads to a downward regulation of the factor, lowering its availability at the site of action. An in vivo transfer of the PDGF-BB into an adenovirus can lead to sustained availability of the factor at the repair site [71], helping tissue regeneration in large defects of the periodontium [59]. Animal studies have shown that recombinant human-derived PDGF can help in vertical and horizontal bone regeneration, and its use has been approved by the FDA for periodontal regeneration [72].

Research indicates that BMP-2 and BMP-7 genes, loaded into an adenoviral vector placed at osseous defect sites help in their repair [68]. Bone morphogenic protein delivery involves the Ad gene transfer of BMP-7, which helps in bone formation and correcting alveolar bone defects. Pluripotent mesenchymal stem cells, used in genetic engineering, placed into osseous defect sites in vivo express BMP-2 and help to induce the formation of bone and blood vessels. Animal studies reveal that bone morphogenetic proteins microencapsulated in polyethylene glycol diacrylate (PEGDA) gels last longer at the defect site, prolonging and distributing their expression [73]. The Cbfa gene, responsible for the expression of BSP (Bone Sialoprotein), which helps in bone regeneration, is being studied for periodontal repair and disease management [74]. RNA-based therapeutics could silence the expression of genes detrimental to tissue regeneration [75]. 

Bruton’s Tyrosine Kinase (BTK) is a mediator of B cell development and plays a role in oncogenic signaling [76]. It is believed to contribute to bone destruction in periodontitis by promoting osteoclasts in inflammatory conditions [77]. Pharmacological inhibitors of BTK have already found application in treating B cell cancers and graft vs. host disease [78,79,80]. Lab studies reveal that pharmacological inhibitors of BTK, such as acalabrutnib, inhibit RANKL and osteoclast differentiation, showing potential in treating experimental periodontitis [81]. Gene therapy has already successfully treated immune disorders caused by derangements in BTK [82,83]. Genetic inhibition of BTK could decrease bone loss through the inactivation of inflammasomes.

A novel application of gene therapy is the use of engineered bacteriophages to combat the effects of periodontal pathogens. Bacteriophages are essentially nucleic acids surrounded by a protein structure, i.e., a virus, that can infect bacteria and destroy it [84]. They can specifically target strains of pathogens involved in biofilm formation and plaque accumulation [85]. Recombinant enzymes of bacteriophages (=lysins) are species-specific with a broad antibacterial activity [86]. Recent studies have shown that engineered lysins are effective in low concentrations against various species such as *Pseudomonas aeruginosa* and *Yersinia pestis* [87,88].

Cell and gene therapy is heralded as a modern approach of limitless possibilities to optimize periodontal healing and regeneration. Yet, challenges remain, such as determining cell sources with clinically effective cell numbers, methods to integrate cells into tissue matrices, augmenting cell and gene expression to achieve functional properties in tissues.

## 4. Antiviral Agents

Antiviral drugs are a class of medications effective against a broad spectrum of viruses. The Epstein-Barr virus (EBV) [89], cytomegalovirus [90], and herpesvirus [91] have been implicated in the etiology of periodontal diseases. Systemic doses of valacyclovir have been found to reduce the viral load of EBV and improve the clinical status of periodontal lesions [92]. Valacyclovir, in conjunction with non-surgical full-mouth debridement, has been shown to improve clinical results of Grade B Stage 2 periodontitis [15,93]. Antiviral drugs such as valacyclovir (Valtrex, GlaxoSmithKline) have been reported to reduce the viral load significantly. It works by suppressing viral multiplication and helps in healing sores to relieve pain and discomfort. Treatment should be started as soon as the symptoms of viral disease, especially herpes, appear [91]. Acyclovir was reported to resolve lesions with no recession in the early stages of herpetic infection [94]. Antiviral therapy has shown substantial improvement in oral lesions in HIV seropositive individuals [95]. Chemotherapeutics can be used against viruses in the lytic stage but not in the latent stage. 

Periodontal tissues may be a possible source of latent viruses [94,95,96]. Viral screening should be considered in cases of unremitting periodontitis [96]. Periodontal therapy consisting of mechanical debridement along with antiviral agents may be indicated in cases with refractory periodontitis. Antiviral mouth rinses and functional nanoparticles are now the subjects of ongoing research as therapeutic agents [97]. Further research is necessary to identify and develop antiviral drugs and unequivocally confirm their efficacy in augmenting mechanical debridement to reduce and suppress the periodontal pathogenic activity.

## 5. Vaccines

The widespread knowledge of cellular and molecular biology has led to the development of vaccines. Vaccines are a cheap and effective tool for improving health, and it has become a central pillar of global health promotion. It is now possible to examine the genome and protein from human pathogens and develop new targets for antimicrobial drugs and vaccines [97]. The immune system has distinct subsets of cells such as helper T cells, memory B cells CD4^+^, and CD8^+^ cells that are stimulated to produce a response against pathogens. This response and the consequent immunological memory protect further assaults from the same pathogen [98]. *Poryphromonas gingivalis*, a Gram-negative bacteria, is one of the most commonly encountered periodontal pathogens. Several studies have targeted the antigenic components of the bacterial fimbriae [99], lipopolysaccharide [100], gingipains [101,102], and its polysaccharide capsule [103] as vaccine targets with varying degrees of success. Whole-cell vaccines of *P. gingivalis* were studied using animal models. While a short-term immune response was elicited, its efficacy as a long-term solution was questionable as no cell-mediated immune responses were triggered [104,105,106]. Long-term immunity was not established, and repeated doses resulted in a lowered response. In comparison, a conjugate vaccine incorporating the polysaccharide capsule antigen effectively reduces oral bone destruction and shows an enhanced immune response across all ages [107]. Synthetic peptides can be used to induce an antibody response. Cell-free protein synthesis systems represent a leap forward in technology, enabling the production of low-cost vaccine antigens. Animal studies use this technology to produce vaccinable targets against *P. gingivalis* protected against bone loss [108]. Plasmid DNA encoding *P. gingivalis* fimbrial gene produces a fimbrial protein in the salivary gland locally rich in Immunoglobulin A, Immunoglobulin G, or their antibodies. These act against *P. gingivalis* in the saliva, reducing their ability to form plaque [109]. The gene hemagglutinin is a virulence factor of *P. gingivalis*. A human monoclonal antibody capable of inhibiting hemagglutinin could help in developing passive immunization against *P. gingivalis* infection [110]. Built-in adjuvant engineered mucosal vaccine targeting the outer membrane protein-like major sheath protein of *F. nucelatum* and *P. gingivalis* appears promising [111]. It successfully induced mucosal and systemic immune responses and prevented periodontal pathology. Research has also focused on antisera against the outer sheath protein of *Treponema denticola* with varied results [112]. Periodontal diseases have numerous causes, thus removing a specific microorganism may not stop the disease from recurring. Challenges such as long-term antibody maintenance, T cell-mediated response, and diverse antigenicities of different microorganisms remain [113]. New vaccine concepts have been proposed to overcome the effects of recent periodontal vaccines that elicited an exaggerated response of T cells and B cells and to overcome their effects.

The search for a safe and effective prophylactic human periodontal vaccine remains ongoing. With further research and analysis, vaccines can be an effective tool in preventing the debilitating effects of periodontal disease on a global scale.

## 6. Proteomics

Proteins play an essential role in almost all biological processes. They are arranged in a specific three-dimensional manner to form an array of molecules. Proteome refers to the entire set of proteins expressed by an organism [114]. Understanding the protein pattern and linkages can help discern the progression of diseases. Proteomics can help analyze protein complements to provide insights into mechanisms of health and disease to usher in breakthroughs and treatments for periodontal diseases [115]. Proteomics examines periodontal pathogens and protein markers to analyze their effect on the physiology of the periodontal ligament. The proteomic analysis helped identify proteins involved in biofilm formation in the proteomes of fusobacterium nucleatum and *P. gingivalis* and highlighted the complex interaction between the bacteria. Recently, salivary visfatin was identified as a potential inflammatory salivary biomarker that could play a role in the pathogenesis of periodontitis [116]. Azurocidin is another potential biomarker candidate for the early detection of gingivitis and periodontitis, identified in the gingival crevicular fluid proteome [117]. These biomarkers can help delineate the variable stages of inflammation, marking the course of infection. 

Inflammatory mediators can also be useful as biomarkers. The bacterial components induce a host response causing the neutrophils and macrophage chemotaxis at the site, inducing the host cells to release cytokines, prostaglandins, tumor necrosis factor, and interleukins. This results in the release of matrix metalloproteins from alveolar bone and polymorphonuclear sites. MMP-8 is of great significance as it is involved in the progression of periodontal diseases. Measurement of MMP-8 can thus help manage periodontal diseases [118].

Shotgun proteomics refers to the analysis and identification of proteins in multiple samples to rapidly generate a profile [119]. This technique has been used to identify salivary biomarkers S100A8 and S100A9 for periodontitis which could be used as a rapid chairside test for screening [120].

*P. gingivalis* releases proteases, known as gingipains, that cleave host protein and contributes to the development of Grade B Stage 2 periodontitis [15]. Proteomic analysis has revealed the heretofore unknown molecular mechanisms behind tissue destruction characterizing the patterns and behavior of various gingipains secreted by the bacteria [121]. These gingipains are valid targets for the prevention of periodontitis [122]. In vitro studies have begun examining a biocompatible gingipain-responsive hydrogel that exerts antibacterial action inhibiting the growth of *P. gingivalis* and supporting periodontal regeneration [123].

Recently, proteomic analysis of the saliva of Grade B Stage 2 periodontitis patients revealed that histatin-1, proline-rich phosphoprotein, thioredoxin, and cystatin-SA were increased in Grade B Stage 2 periodontitis [15]. These differences in salivary protein profiles can be used as disease indicators and help in the diagnosis [124]. Proteomic profiling of gingival crevicular fluid for disease biomarkers and osteogenic differentiation potential in periodontal ligament stem cells has provided new avenues for diagnosis and periodontal regeneration [125,126]. Evaluations and comparisons of the proteome profile could highlight alterations in diseased and healthy conditions and drive the discovery of prospective biomarkers. Ongoing trials are using proteomics to examine immune response after treatment and characterize the periodontal condition in implantitis and systemic diseases such as diabetes mellitus and obesity [127,128,129,130]. Proteomics presents a leap ahead in periodontal diagnostics and is valuable for the discovery and validation of periodontal health and disease biomarkers.

## 7. Nanoparticles

Nanotechnology refers to the science of exploiting and manipulating supramolecular materials on a nanoscale. Advancing nanotechnology could have an enormous impact on the treatment of periodontal diseases. Nanometric dimensions allow for targeted drug delivery to tissues that cannot be reached by conventional drug delivery systems, enabling the controlled release of antibiotics and anti-inflammatory drugs to address the aggressions of periodontal pathogens or modulating host cell responses [16]. Nanotechnology-based materials have immense applications in localized drug delivery, guided tissue regeneration, and bone regeneration. Hydrogels composed of nanofibers and κ-carrageenan oligosaccharides nanoparticles have been found to have strong antibacterial activity against several periodontal pathogens and decreased biofilm formation. These hydrogels also exhibited anti-inflammatory activity by reducing reactive oxygen species formation and modulating cytokine production in gingival fibroblasts [131]. Nanoparticles can be used to support polymer-drug conjugates such as polysaccharides to maintain a constant high concentration of the drug in a periodontal pocket [132]. 

Recent research has examined the use of nanocomposites for sustained delivery of drugs such as triclosan [133], tetracycline [134], minocycline [135], resveratrol [136] tinidazole [137], doxycycline [138], and metronidazole [139]. These nanocarriers protect the drug from degradation, improve the solubility, and control the release kinetics of the drugs. The nanoparticles provide stability and sustained release at a local site. This local availability is important as systemic administration of antibiotics is associated with increased hepatic and renal toxicity due to ineffective penetration and uptake [140]. Several polymers can also be used as Nano drug delivery systems such as chitosan [141], poly lactic-co-glycolic acid copolymer [142], and ethyl vinyl acetate [143]. Silver nanoparticles enclosed in a chitosan matrix showed the ability to reduce S. mutans biofilm formation [144]. Nanospheres and nanocapsules loaded with chlorhexidine showed a controlled release over a large surface area at different pH with the ability to infiltrate the gingival tissue subgingivally. The formulation decreased dental plaque better than commercially available mouth rinses [145].

Periodontal regeneration is the overarching principle of periodontal therapy. Emergent nanotechnology has brought this significant challenge within grasp. Recent research has demonstrated that nanocomposite scaffolds derived from electrospun nanofibers can be blended with growth factors and proteins to form matrices that can modify the local microenvironment and encourage periodontal regeneration [146]. Hydroxyapatite and silver nanoparticles incorporated into nanofiber scaffolds show enhanced antibacterial and bone regeneration ability with increased cellular viability and a desirable degradation profile, dissolving with time [147].

Electrospun nanofibers have immense potential in the field of tissue engineering and periodontics. Electrostatic forces are used to create a mesh of nanofibers from single or multiple polymers [148]. Their ability to mimic natural extracellular matrix, ease of fabrication, customizable pore size, and shape make them ideal for enhancing tissue regeneration [149]. These nanofibers allow for incorporating biologically active molecules into the core component, allowing for controlled drug delivery and guided tissue and bone regeneration [150]. Recent studies have confirmed their biological functions in the form of cellulose nanofibrils and membranes using precursors such as polylactic acid (PLA), polyglycolic acid (PGA), PCL (polycaprolactone) [151], and enamel matrix derivatives [152] for bone and periodontal tissue regeneration. Biodegradable natural polymers such as collagen, fibrinogen, chitin, and glycosaminoglycans can also be used to create resorbable membranes. A mixture of natural and synthetic polymers provides the best of both worlds with better mechanical and biological properties [153].

Nanoparticles offer unique programmable properties such as bio-adhesiveness, stimuli-responsive behavior, and customizable structures that can be used to tackle periodontal diseases. Nanotechnology in the form of nanofiber membranes and electrospun fibers will improve treatment outcomes and herald a new era of periodontal regenerative medicine.

## 8. Conclusions

Periodontal disease has numerous etiopathogenesis and diverse impacts. Therefore, the measures taken to combat the disease must be multifaceted for long-term periodontal stability. This paper summarizes the pharmaceutical, gene-therapy, and nanotechnology targets for future periodontal therapy. Ultimately, therapeutic success depends on managing periodontitis through an integrative approach to resolve inflammation and restore homeostasis.
